# The Influence of Type 1 Diabetes Mellitus on Dental Caries and Salivary Composition

**DOI:** 10.1155/2018/5780916

**Published:** 2018-10-02

**Authors:** Lulëjeta Ferizi, Fatmir Dragidella, Lidvana Spahiu, Agim Begzati, Vjosa Kotori

**Affiliations:** ^1^Department of Pediatric Dentistry, School of Dentistry, Medical Faculty, University of Prishtina, Prishtina, Kosovo; ^2^Department of Periodontology and Oral Medicine, School of Dentistry, Medical Faculty, University of Prishtina, Prishtina, Kosovo; ^3^Department of Endocrinology, Pediatric Clinic, School of Medicine, Medical Faculty, University of Prishtina, Prishtina, Kosovo

## Abstract

Diabetes mellitus is the most common chronic disease that affects the oral health. The aim of the study is to evaluate the dental caries, salivary flow rate, buffer capacity, and Lactobacilli in saliva in children with type 1 diabetes mellitus compared to the control group. *Methods.* The sample consisted of 160 children of 10 to 15 years divided into two groups: 80 children with type 1 diabetes mellitus and 80 children as a control group. Dental caries was assessed using the DMFT index for permanent dentition. Stimulated saliva was collected among all children. Salivary flow rate and buffer capacity were measured, and the colonies of *Lactobacillus* in saliva were determined. The observed children have answered a number of questions related to their dental visits and parents' education. The data obtained from each group were compared statistically using the chi-square test and Mann–Whitney *U*-test. The significant level was set at *p* < 0.05. *Results*. DMFT in children with type 1 diabetes was significantly higher than that in the control group (*p* < 0.001). Diabetic children have a low level of stimulated salivary flow rate compared to control children (0.86 ± 0.16 and 1.10 ± 0.14). The buffer capacity showed statistically significant differences between children with type 1 diabetes and control group (*p* < 0.001). Also, children with type 1 diabetes had a higher count and a higher risk of *Lactobacillus* compared to the control group (*p* < 0.05 and *p* < 0.001). *Conclusion*. The findings we obtained showed that type 1 diabetes mellitus has an important part in children's oral health. It appears that children with type 1 diabetes are exposed to a higher risk for caries and oral health than nondiabetic children.

## 1. Introduction

Diabetes mellitus (DM) is a common chronic disease that leads to hyperglycemia [1–3]. It is classified into four general categories: type 1, in which the pancreas *β*-cells lose their capacity to produce insulin; type 2, in which a defect in the *β*-cells or a reduction in tissue sensitivity to insulin is necessary for disease manifestation; gestational diabetes, defined as any degree of glucose intolerance with onset or first recognition during pregnancy; and specific types of diabetes due to other causes, e.g., monogenic diabetes syndromes (such as neonatal diabetes and maturity-onset diabetes of the young (MODY)), diseases of the exocrine pancreas (such as cystic fibrosis), and drug- or chemical-induced diabetes (such as with glucocorticoid use, in the treatment of HIV/AIDS, or after organ transplantation) [1].

The oral cavity structure can be affected by diabetes, which may result in several complications including dental caries, periodontal disease, oral mucosal diseases, and saliva dysfunction that have a significant effect on the quality of life of diabetic patients. Also, untreated oral diseases may increase the risk of poor metabolic control [4]. The relationship between diabetes and dental caries has received the attention of researchers because both of the diseases are associated with carbohydrates. The insulin deficiency in diabetes may lead to hyposalivation and elevated salivary glucose levels, which may put diabetic patients at a high risk of caries development [5]. Saliva composition is an important factor in determining the prevalence of caries and oral health. It maintains the integrity of oral tissues, provides protection against immunologic bacterial, fungal, and viral infections [6], and controls the equilibrium between demineralization and remineralization in a cariogenic environment. Also, salivary buffers can stabilize pH in plaque, thus preventing demineralization of enamel [7–9]. Patients with diabetes have been reported to complain of dry mouth and salivary dysfunction leading to a reduction of salivary flow rate, lower buffer capacity, increased risk for dental caries, and bacterial infections [10].

Increasing the level of glucose in saliva affects the activity of microorganisms. *Streptococcus mutans* and *Lactobacillus* are considered to be related to caries and are the most cariogenic bacteria [11] because they have the ability to create a low pH environment and progression of caries [12]. Research studies show that *Streptococcus mutans* and *Lactobacillus* found in stimulated saliva explain better the development of caries than *Streptococcus mutans* and *Lactobacillus* found in plaque [13, 14]. For this reason, the combined analysis of dental caries, salivary components, and bacterial pathogens in saliva is a powerful method of following the oral diseases in children with type 1 diabetes mellitus [15].

The parents' role is very important in relation to oral health because they are the main caregivers of their children's oral health [16]. The studies show that the parents of children with diabetes are often careless about untreated dental caries in their children and not conscious enough on the importance of their oral health and its influence in diabetes [17, 18].

The aim of the study was to assess the dental caries, salivary flow rate, buffer capacity, and bacterial count of *Lactobacillus* in saliva between children with type 1 diabetes mellitus and control group.

## 2. Materials and Methods

### 2.1. Study Sample

The study was conducted in 160 children, including 80 children with type 1 diabetes mellitus aged 10–15 years, who were attending the Pediatric Clinic at University Medical Centre of Prishtina, Republic of Kosovo. All diabetic children were treated with insulin but not with any other therapy within the last month. The control group aged 10–15 years included 80 healthy children with absence of active diseases and no history of drug therapy within the previous month.

### 2.2. Clinical and Microbiological Procedures

All children were examined by a researcher at the Department of Pediatric Dentistry, University Dentistry Clinical Centre of Kosovo (UDCCK). Before children's examination, an informed consent was received from their parents. The clinical dental health status was measured using the Decayed, Missing and Filled Teeth (DMFT) Index for permanent teeth according to the WHO caries diagnostic criteria for epidemiological studies [19].

The results of examination of the saliva were compared with the results of a control group of healthy children, corresponding in number and age of studied diabetic children. The test for evaluation of saliva included salivary flow rate, buffer capacity, and colonies of *Lactobacillus* in stimulated saliva. For at least one hour before the test is conducted, patients should neither eat nor drink anything. Each subject was given a piece of paraffin pellet and asked to chew the paraffin and to expectorate the stimulated saliva into the sterile container. Flow rate (5 min production) was defined as the volume of saliva secreted per min. The CRT buffer is used to determine the buffer capacity of saliva by means of a test strip featuring a special indicator system (Ivoclar Vivadent, Liechtenstein). Pipetted stimulated saliva from the container was dropped in each of the three fields of the strip test. The color of the field changed immediately, but the results were assessed after the expiration of the manufacturer's reaction time (5 minutes) in the color scale. Blue indicates a high buffer capacity, green indicates a medium buffer capacity, and yellow color indicates a low buffer capacity of saliva. The CRT buffer enables the buffering capacity of saliva to be quickly and efficiently determined. For the microbial count identification, saliva was used instead of dental plaque because the saliva is sufficiently representative of the available microflora in the oral cavity. The presence of *Lactobacillus* was determined using the CRT bacteria test (Ivoclar Vivadent, Liechtenstein) on the saliva previously stimulated by chewing paraffin. Bacterial counts were recorded as colony-forming units per milliliter (CFU/mL) of saliva. The number of *Lactobacillus* colonies was graded as follows: Class 1 (none detected), Class 2 (10^2^–10^3^ CFU/mL), Class 3 (10^4^–10^5^ CFU/mL), and Class 4 (CFU ≥ 10^5^/mL), according to the manufacturers' scoring card.

### 2.3. Questionnaire

All study participants were asked to fill in a prepared questionnaire during their visit to the dental clinic. The questions were answered by the children under the parental supervision.

The questionnaire included sections related to the frequency of dental visits and parents' education. The parents' education level was categorized into those who completed low-level education (primary school), middle-level education (secondary school), and high-level education (university).

### 2.4. Ethical Aspects

This study was approved by the Ethical Committee of Medical Faculty of the University of Prishtina, Kosovo, with Reference Number 4000/2016. Written informed consent was obtained from parents of children that were included in this study.

### 2.5. Data Analysis

The statistical analysis was carried out using MS Excel (Microsoft Corporation, Redmond, WA, USA) and SPSS 17 (SPSS Inc., Chicago, Illinois, USA) software. Percentages were compared by using the chi-square test. The difference in the values of D, M, F, and DMFT index for permanent teeth, between type 1 diabetes mellitus and healthy children, was tested using the Mann–Whitney *U*-test. Differences were set to be statistically significant at *p* < 0.05.

## 3. Results

Children included in this study were divided into two groups as children with and without type 1 diabetes mellitus. The results shown in Table 1 refer to the age and buffer capacity among the two groups of children. No significant difference between two groups with respect to the age of children was found (*p* > 0.05). Regarding buffer capacity, children with type 1 diabetes have a low buffer capacity and a medium buffer capacity (45.0% and 33.7%), whereas children from the control group have a high buffer capacity and a medium buffer capacity (39.4% and 31.3%) (Table 1).

The difference between the DMFT index of diabetic children and nondiabetic children is presented in Table 2. The component D was significantly higher in diabetic children (*p* < 0.001), whereas component F was higher in the control group (*p* < 0.001). No significant difference between groups related to component M (*p* > 0.05) was found. In total, the DMFT index of children with type 1 diabetes mellitus was higher (*p* < 0.001) compared to the DMFT index of nondiabetic children.

The average and standard deviation of salivary flow rate in children with type 1 diabetes mellitus are lower (0.86 ± 0.16 mL/min) than those of children in the control group (1.10 ± 0.14 mL/min) (Table 3).

The results related to *Lactobacillus* in both groups of children are shown in Table 4. Children with type 1 diabetes have significantly lower levels of colonies of *Lactobacillus* in Class 1 and Class 2 (0% and 27.5%) than the control group (21.3% and 51.3%). The colonies of Class 3 (10^4^–10^5^ CFU/mL) tend to be similar in both groups, in terms of *Lactobacillus* (25.0% and 23.8%), but regarding Class 4 of *Lactobacillus*, children with type 1 diabetes have higher levels of colonies of *Lactobacillus* than the control group (47.5% and 3.8%). Type 1 diabetes children are predisposed to have a higher caries risk of *Lactobacillus* than the control group. Low risk for caries was found in 27.5% of children with type 1 diabetes mellitus and 72.5% of the control group, whereas high risk for caries was significantly higher in children with type 1 diabetes (72.5%) than in the control group (27.5%) (Table 4).

As shown in Table 5 regarding dental visits, there is a significant difference between groups. The majority of children with type 1 diabetes visited the dentist only when necessary, whereas children from the control group visited dentist once a year (*p* < 0.001). Related to the parents' education, children with type 1 diabetes mostly have medium and low levels of parents' education, with a difference from the control group, which dominates with the medium and higher levels of parents' education (*p* < 0.001).

## 4. Discussion

The prevalence of dental caries and its burden on the general population are of significant public health interest. Therefore, it is important to identify patients who may be at a high risk of dental caries and oral disease [20]. Diabetes mellitus may increase one's susceptibility to dental caries. In addition, people with diabetes are also more prone to infections, including dental abscesses that are a result of progressive dental caries [5].

The results from the present study show that oral health of children with type 1 diabetes in Kosovo is a serious health problem. Previous studies conducted in Kosovo regarding dental caries among children and healthy adults reported high scores of dental caries [21–24]. In our study, the prevalence of dental caries was significantly higher among diabetic patients than nondiabetic patients. Several authors have reported similar findings [25–27], others reported low prevalence of dental caries among diabetics [28, 29], and some authors did not find any significant difference in the DMFT index between type 1 diabetic children and control group [30, 31]. Increased risk of dental caries would be related to certain factors such as poor oral hygiene, rare dental visits, and lack of metabolic control of diabetes.

Most of the studies have shown that patients with diabetes manifested low salivary flow rates, high levels of glucose in saliva [32, 33], and complaints of dry mouth [28]. Salivary flow rates are reduced in diabetic patients, and this may increase the sensitivity to oral infections, especially when there is a poor metabolic control of the disease. The results of this study showed a significant decrease of stimulated salivary flow rate and buffer capacity in diabetic patients when compared with nondiabetic children. A similar finding was also reported by other studies [34, 35]. Salivary flow rate in the present study was decreased in diabetic patients, but this finding was in disagreement with studies by Edblad et al. [36], Belazi et al. [37], and Canepari et al. [38]. They showed no difference in the salivary flow rate between diabetic and control subjects. Among several reasons which contribute to the decreased salivary flow rate in diabetes is hyperglycemia and glucosuria which cause a lower secretion of saliva. Also, if the diabetes is uncontrolled, these changes are more expressive [39].

In saliva, there are three major systems contributing to the buffer capacity: bicarbonate, phosphate, and protein buffer systems. The buffer capacity of saliva is an important factor, which has an important role in the maintenance of salivary pH and in dental remineralization. It correlates with salivary flow rate, and if any factor that decreases the salivary flow rate, it declines also its buffer capacity and increases the risk of caries development [40–42]. In our study, there was a significant difference in the mean salivary buffering capacity among study groups (*p* < 0.001). The results obtained are in accordance with the study performed by Aral et al. [43]. In their study, the authors found that the percentage of individuals with low salivary buffer capacity was highest in the diabetic group and lowest in the control group. However, other studies have shown no considerable difference between children with type 1 diabetes mellitus and control group related to buffer capacity [36, 38].

The buffer capacity also was studied by Saes Busato et al. [44], among adolescents with type 1 diabetes mellitus (14–19 years) and nondiabetic group. The adolescents in the type 1 diabetes mellitus group were evaluated at a baseline (*T*_0_) and after 15 months (*T*_1_), and those in the nondiabetic group were only evaluated at baseline (*T*_0_). The salivary buffering capacity was slightly reduced after 15 months in adolescents with type 1 diabetes mellitus; at *T*_0_, it was 4.8, and at *T*_1_, it was 3.9. The study suggests that the hyposalivation and duration of the disease associate with a reduction in the buffer capacity in children with type 1 diabetes.

Most of the studies in diabetic children have analyzed the presence of *Streptococcus mutans* and *Lactobacillus* in the saliva. But in our study, the main focus was on the colonies of *Lactobacillus*, and the results showed a correlation between different levels of *Lactobacillus* in saliva; high levels of *Lactobacillus* were found in classes with a higher risk for caries. The similar results related to salivary Lactobacilli have been reported in other studies conducted by De Tove et al. [45] and Al-Khayoun et al. [46]. The authors found high levels of *Lactobacillus* in saliva in children with type 1 diabetes and evaluated that the poor metabolic control of diabetes had a significant effect on the *Lactobacillus* level in the saliva. Unlike our study, other studies reported no differences between the levels of *Lactobacillus* among children with type 1 diabetes and control group [18, 36, 47].

Twetman et al. [48] evaluated the quantitative distribution of *Streptococcus mutans* and *Lactobacillus* in saliva of type 1 diabetic children (aged 4–19) compared to healthy children regarding the metabolic control of the disease. They found low levels of *Lactobacillus* in the diabetic children which correlated with glucose concentration in saliva. Their findings suggest that the dietary treatment of children with type 1 diabetes reduced the number of Lactobacilli in saliva.

Although the results in our study report that children with type 1 diabetes have a higher caries risk of *Lactobacillus*, López Del Valle et al. [47] found no difference between type 1 diabetic children and control group regarding the caries risk of *Lactobacillus*. Diminished salivary flow is a suitable environment for the establishment of *Streptococcus mutans* and *Lactobacillus* in the oral cavity of diabetic patients, especially among the uncontrolled diabetes group. High levels of these bacteria in saliva can be considered an indicator of a cariogenic environment in the mouths of diabetes subjects. *Streptococcus mutans* is the main bacterium responsible for the occurrence of dental caries, whereas *Lactobacillus* is more related to the progression of caries due to its ability to adhere to the tooth surface [47].

The present study reports that the children with type 1 diabetes visited the dentist only when necessary, and our results were consistent with the study conducted by Tagelsir et al. [49] where children with type 1 diabetes rarely visited the dentist. Unlike our study, other studies have shown that children with type 1 diabetes visited the dentists at least once a year [50, 51]. Surprisingly, Al-Khabbaz et al. [52] found that only 24% of the diabetic children had their first dental visit before the age of 4 years, and a large number of them (44%) had never visited the dentist before. Apparently, the current health service in Kosovo provides free access to dental care to all Kosovo's children up to 15 years, and parents should be encouraged to use these services to maintain the oral health of their children with type 1 diabetes mellitus.

Parents' education and the impact of family is a well-recognized risk factor for caries and metabolic control in children with type 1 diabetes mellitus. The level of parents' education regarding diabetic children in our study was medium and low, whereas the control group dominated with the medium and higher levels of parents' education. The findings of our study are similar to those of other studies related to the level of parents' education [18, 53]. In disparity to the results of our study, Siudikiene et al. [54] in their study found that a mother's education level was not an important predictor of high caries experience. Parents of children with type 1 diabetes have a lack of sufficient knowledge on their children's oral health and its influence on general health and also on metabolic control of diabetes. Therefore, parents' education and their active involvement in their child's diabetes self-management are crucial tools to achieve the desired goals.

### 4.1. Study Limitation

The researcher did not collect the information regarding the well-controlled and poorly controlled diabetes which may affect the dental caries and bacterial count. The authors may suggest further studies.

## 5. Conclusion

Diabetes is a risk factor for oral health complications. The findings of this study showed that children with type 1 diabetes mellitus exhibited significantly more dental caries, low salivary flow rate and buffer capacity, and higher count of *Lactobacillus* than healthy children. In addition, diabetic children must do regular dental visits. Parents also play a major role in their children's oral health, and the dentist and pediatrician should inform them of the importance of their children's oral health and routine dental checkups.

## Figures and Tables

**Table 1:** The differences in age and buffer capacity between groups.

Age	Type 1 DM		Control group		Total		Test
	*N*	%	*N*	%	*N*	%	
10 years	15	18.8	15	18.8	30	18.8	Chi = 0.37; *p* > 0.05
11 years	11	13.8	12	15.0	23	14.4	
12 years	14	17.5	16	20.0	30	18.8	
13 years	13	16.3	12	15.0	25	15.6	
14 years	13	16.3	13	16.3	26	16.3	
15 years	14	17.5	12	15.0	26	16.3	
Total	80	100.0	80	100.0	160	100.0	

*Buffer capacity*							
High	17	21.3	46	57.5	63	39.4	Chi = 26.97; *p* < 0.001
Medium	27	33.7	23	28.8	50	31.3	
Low	36	45.0	11	13.7	47	29.3	

**Table 2:** Difference in D, M, F, and DMFT index between groups.

Variable	Rank sum of type 1 DM	Rank sum of the control group	*U*	*Z* adjusted	*p* level	Valid *N* of type 1 DM	Valid *N* of the control group	*p* value
D	8563.00	4317.00	1077.00	7.32	**0.000**	80	80	*p* < 0.001
M	6102.50	6777.50	2862.50	−1.33	0.18	80	80	*p* > 0.05
F	5490.00	7390.00	2250.00	−3.36	**0.000**	80	80	*p* < 0.001
DMFT index	7756.00	5124.00	1884.00	4.53	**0.000**	80	80	*p* < 0.001

**Table 3:** The average and standard deviation of salivary flow rate.

Variable	Valid *N*	Mean ± SD	Confidence − 95.00%	Confidence + 95.00%	Minimum	Maximum
Stimulated salivary flow rate/type 1 DM	80	0.86 ± 0.16	0.82	0.89	0.50	1.30
Stimulated salivary flow rate/control group	80	1.10 ± 0.14	1.07	1.13	0.80	1.40

**Table 4:** General and specific distribution of *Lactobacillus* between groups.

*Lactobacillus* (LB)	Type 1 DM		Control group		Total		Test
	*N*	%	*N*	%	*N*	%	
Class 1 (not detected)	0	0	17	21.3	17	10.6	Chi = 7.33; *p* < 0.05
Class 2 (10^2^–10^3^ CFU/mL)	22	27.5	41	51.3	63	39.4	
Class 3 (10^4^–10^5^ CFU/mL)	20	25.0	19	23.8	39	24.4	
Class 4 (>10^5^ CFU/mL)	38	47.5	3	3.8	41	25.6	

*Lactobacillus values in CFU/mL saliva (caries risk test for LB)*							
Low (<10^5^ (1 and 2))	22	27.5	58	72.5	80	50.0	Chi = 20.73; *p* < 0.001
High (≥10^5^ (3 and 4))	58	72.5	22	27.5	80	50.0	

**Table 5:** Dental visits and parents' education between groups.

Dental visits	Type 1 DM		Control group		Total		Test
	*N*	%	*N*	%	*N*	%	
Once in 6 months	12	15.0	17	21.3	29	18.1	Chi = 20.73; *p* < 0.001
Once a year	19	23.8	42	52.5	61	38.1	
Only when necessary	49	61.3	21	26.3	70	43.8	

*Father's education*							
Low level	19	23.8	5	6.3	24	15.0	Chi = 27.22; *p* < 0.001
Medium level	56	70.0	45	56.3	101	63.1	
High level	5	6.3	30	37.5	35	21.9	

*Mother's education*							
Low level	46	57.5	22	27.5	68	42.5	Chi = 24.94; *p* < 0.001
Medium level	31	38.8	34	42.5	65	40.6	
High level	3	3.8	24	30.0	27	16.9	

## Data Availability

The data used to support the findings of this study are available from the corresponding author upon request. The corresponding author possesses data and may make data available upon request.
